# Supplementary material for: Effect of exercise based interventions on sleep and circadian rhythm in cancer survivors—a systematic review and meta-analysis
Source: PeerJ. 2024 Mar 8;12:e17053. doi: 10.7717/peerj.17053 (PMC10926908; doi:10.7717/peerj.17053)
Supplement: Supplemental Information 8 [file peerj-12-17053-s008.docx]

**S-2a** Summary of outcome measures used for sleep quality.

| **Study** | **Self-reported** | | | | | | **Accelerometery** | | | | | | | | | |
| --- | --- | --- | --- | --- | --- | --- | --- | --- | --- | --- | --- | --- | --- | --- | --- | --- |
|  | PSQI | GSDS | ISI | SDS | EORTC  QLQ-C30 | 11-point NRS | AWT | AST | Movement in sleep | Immobility time | SE | TST | WASO | SOL | Mean activity score | MFI |
|  | **AEROBIC TRAINING** | | | | | | | | | | | | | | | |
| Payne et. al, 2008 | ✔ |  |  |  |  |  | ✔ | ✔ | ✔ |  | ✔ |  |  |  |  |  |
| Dodd et al.,2010 |  | ✔ |  |  |  |  |  |  |  |  |  |  |  |  |  |  |
| Tang et al., 2010 | ✔ |  |  |  |  |  |  |  |  |  |  |  |  |  |  |  |
| Wang et al., 2011 | ✔ |  |  |  |  |  |  |  |  |  |  |  |  |  |  |  |
| Courneya et al.,2012 | ✔ |  |  |  |  |  |  |  |  |  |  |  |  |  |  |  |
| Cho et al., 2012 |  | ✔ |  |  |  |  |  |  |  |  |  |  |  |  |  |  |
| Wenzel et al., 2013 | ✔ |  |  |  |  |  |  |  |  |  |  |  |  |  |  |  |
| Naraphong et al., 2015 |  | ✔ |  |  |  |  |  |  |  |  |  |  |  |  |  |  |
| Chen et al., 2016 | ✔ |  |  |  |  |  |  |  |  |  | ✔ | ✔ | ✔ | ✔ |  |  |
| Roveda et al., 2017 |  |  |  |  |  |  | ✔ | ✔ |  | ✔ | ✔ |  |  | ✔ | ✔ | ✔ |
| Mercier et al., 2018 | ✔ |  | ✔ |  |  |  |  |  |  |  | ✔ | ✔ | ✔ | ✔ |  |  |
| Khoirrunnisa et al., 2019 |  |  |  | ✔ |  |  |  |  |  |  |  |  |  |  |  |  |
|  | **RESISTANCE TRAINING** | | | | | | | | | | | | | | | |
| Steinndorf et. al, 2017 |  |  |  |  | ✔ |  |  |  |  |  |  |  |  |  |  |  |
|  | **COMBINED TRAINING** | | | | | | | | | | | | | | | |
| Sprod et. al, 2010 | ✔ |  |  |  |  |  |  |  |  |  |  |  |  |  |  |  |
| Chivelle et. al, 2014 |  |  |  |  |  | ✔ |  |  |  |  |  |  |  |  |  |  |
| Kampshoff et al.,2015 | ✔ |  |  |  |  |  |  |  |  |  |  |  |  |  |  |  |
| Rogers et. al, 2015 | ✔ |  |  |  |  |  |  |  |  |  | ✔ |  |  | ✔ |  |  |
| Coleman et al., 2012 |  |  |  |  |  |  |  |  |  |  | ✔ |  |  |  |  |  |
| Courneya et al., 2014 | ✔ |  |  |  |  |  |  |  |  |  |  |  |  |  |  |  |
|  | **PHYSICAL ACTIVITY** | | | | | | | | | | | | | | | |
| Rogers et al.,2009 | ✔ |  |  |  |  |  |  |  |  |  |  |  |  |  |  |  |
| Donnelly et al., 2011 | ✔ |  |  |  |  |  |  |  |  |  |  |  |  |  |  |  |
| Rogers et al., 2013 | ✔ |  |  |  |  |  |  |  |  |  | ✔ |  |  | ✔ |  |  |
| Rogers et al., 2017 | ✔ |  |  |  |  |  |  |  |  |  | ✔ |  |  | ✔ |  |  |
| Li et al., 2022 | ✔ |  |  |  |  |  |  |  |  |  |  |  |  |  |  |  |
|  | **YOGA** | | | | | | | | | | | | | | | |
| Cohen et al., 2004 | ✔ |  |  |  |  |  |  |  |  |  |  |  |  |  |  |  |
| Raghavendra et al., 2009 |  |  |  |  |  |  |  |  |  |  |  |  |  |  |  |  |
| Mustain et al.,2013 | ✔ |  |  |  |  |  |  |  |  |  |  |  |  |  |  |  |
| Chandwani et al., 2014 | ✔ |  |  |  |  |  |  |  |  |  |  |  |  |  |  |  |
| Cramer et al., 2016 | ✔ |  |  |  |  |  |  |  |  |  |  |  |  |  |  |  |
| Taylor et al., 2018 |  |  | ✔ |  |  |  |  |  |  |  |  |  |  |  |  |  |
| Chaoul et al., 2018 | ✔ |  |  |  |  |  |  |  |  |  | ✔ | ✔ | ✔ | ✔ |  |  |
| Huberty et. al, 2019 |  |  |  | ✔ |  |  |  |  |  |  |  |  |  |  |  |  |
|  | **TAI CHI** | | | | | | | | | | | | | | | |
| Larkey et. al, 2015 | ✔ |  |  |  |  |  |  |  |  |  |  |  |  |  |  |  |
| Irwin et. al, 2017 | ✔ |  |  |  |  |  |  |  |  |  | ✔ |  | ✔ | ✔ |  |  |
| McQuade et.al.,2017 | ✔ |  |  |  |  |  |  |  |  |  |  |  |  |  |  |  |
| Lu et. al.,2019 | ✔ |  |  |  |  |  |  |  |  |  |  |  |  |  |  |  |

*AST- Actual Sleep Time; AWT-Actual Wake time; GSDS-General Sleep Disturbance Scale; EORTCQLQ-C30 insomnia subscale (sleep); ISI-Insomnia Severity Index; PSQI- Pittsberg Sleep Quality Index SDS-Sleep Disturbance Scale; SE-Sleep Efficiency; SOL-Sleep Onset Latency; TST- Total Sleep time; WASO- Wake after sleep onset*
