# Supplementary material for: Effect of exercise based interventions on sleep and circadian rhythm in cancer survivors—a systematic review and meta-analysis
Source: PeerJ. 2024 Mar 8;12:e17053. doi: 10.7717/peerj.17053 (PMC10926908; doi:10.7717/peerj.17053)
Supplement: Supplemental Information 9 [file peerj-12-17053-s009.docx]

| **Study** | **Circadian rhythm (accelerometery)** | | **Circadian rhythms (biomarkers)** | | |
| --- | --- | --- | --- | --- | --- |
|  | R24, I<O | MESOR | Serum Cortisol | Serum serotonin | Salivary cortisol |
|  | **AEROBIC TRAINING** | | | | |
| Payne et. al, 2008 |  |  | ✔ | ✔ |  |
| Chen et al., 2016 | ✔ |  |  |  |  |
| Roveda et al., 2017 |  | ✔ |  |  |  |
|  | **YOGA** | | | | |
| Raghavendra et al., 2009 |  |  |  |  | ✔ |
| Chandwani et al., 2014 |  |  |  |  | ✔ |

**S-2b** Summary of outcome measures used for circadian rhythm.

*I < O- in-bed less than out-of-bed dichotomy index; r24- 24 hours autocorrelation coefficient.*
