# Supplementary material for: Effect of exercise based interventions on sleep and circadian rhythm in cancer survivors—a systematic review and meta-analysis
Source: PeerJ. 2024 Mar 8;12:e17053. doi: 10.7717/peerj.17053 (PMC10926908; doi:10.7717/peerj.17053)
Supplement: Supplemental Information 10 [file peerj-12-17053-s010.docx]

**S-3** Summary of the methods and intervention for the exercise studies.

| Author, Year | Samples | | Treatment | Intervention | | | | | | | |
| --- | --- | --- | --- | --- | --- | --- | --- | --- | --- | --- | --- |
|  | Cancer type and stage | Age, Gender, sample size |  | Type and details | | | Intensity | | Time duration | | Frequency |
| AEROBIC TRAINING | | | | | | | | | | | |
| Payne et. al, 2008 | Breast Ca | >55years  20 (F)  20: 10 (EX)+ 10 (UC) | Hormonal therapy | EX- Home-based walking program  UC- gift card was given | | | Moderate | | 20 min/ session | | 4 times/ week for 14 weeks |
| Dodd et al., 2010 | breast, colorectal, or ovarian cancer | >18years  119 (F)  44 (EE) + 36 (CE) + 39 (CC) | During and after Chemotherapy | EE  cardiovascular/aerobic exercise e.g., walking, jogging, or  bicycling  CE- Same exercise after completion of treatment  CC: standard care/usual activities | | | moderate (training heart rate corresponding to 60–  80%  VO2peak) | | 20–30 min/session | | 3–5 times per week for 1 year |
| Tang et al., 2010 | Mixed | >18years (17M +54 F)  37 (EG) + 35 (CG) | No treatment (50), Chemotherapy (10), Radiotherapy (7), Hormonal therapy (4) | EG: Home based brisk walking with recording of the duration of walk, the RPE,  comments or difficulties. Every 2 weeks, diaries were returned to the researcher.  CG: usual activities | | | moderate  RPE- 11-13 | | 30min/session | | 3days/week for 8 weeks |
| Wang et al., 2011 | stage I or stage II breast cancer | 18 to 72 years, (0M+ 72F)  35 (EG) + 37 (UG) | Surgery and Chemotherapy | EG: walking program based on Bandura’s Self-efficacy Theory  Weekly recording of exercise log, weekly phone meeting between participant and researcher.  UG: Usual care | | | 40% to 60%  heart rate maximum (HRmax)  or the modified Borg Scale between 0.5 and 2 | | at least 30 minutes per session or the accumulation  of 10-minute sessions to reach 30 minutes | | 3-5/week for 6 weeks |
| Courneya et al., 2012 | Hodgkin lymphoma  (HL) or non–Hodgkin lymphoma (NHL), / no evidence of disease, stage I/II, and III/IV | >18yrs (69M + 48F)  UC (60) + AET (57) | Chemotherapy (53), No treatment (64) | AET: supervised cycle ergometer  UC: asked not to increase  their exercise from baseline | | | 60-75% of VO2peak  (Increased from 60% to 5% every week) | | 15-20min (1st week) to 40-45min (by 9th week)  (Increase by 5min/week until 45min) | | 3days/week for 12 weeks |
| Cho et al.,2012 | breast, colorectal, or ovarian cancer/ stage 1-3 | ≥ 18 years (0M + 119F)  exercisers (52) + non exercisers (65) | Sequential chemotherapy then radiation therapy (59) | EE: received their exercise prescription throughout the study period (both during & after cancer treatment)  CE: received exercise prescription after  having completed cancer treatment  CC: usual care  Exercises included- walking, jogging/ running, swimming, and cycling | | | moderate intensity | | 20min/session | | 3days/week for 1 year |
| Wenzel et al.,2013 | stage I–III solid malignant  tumor | >21years (77M +49F)  126: EX (68) + UC (58) | Chemotherapy (44) Radiotherapy (66), Both (9), Brachytherapy (7) | EX: Pedometer based unsupervised brisk walking program  UC: Instructed  to maintain their usual physical activity | | | 50-70% of HRmax | | 20-30min + 5min warm up + 5min cool down | | 5days/week throughout the treatment course (ranged from 5-35 weeks) |
| Naraphong et al., 2015 | Breast Cancer Stage I–IIIa | 18-60 Yrs. (23F +0M)  23 (11EG + 12CG) | adjuvant chemotherapy | EG: modified the exercise intervention to be culturally relevant Thai physical activities + walking  UG: usual care and weekly telephone  calls | | | low-intensity to moderate-intensity  exercise <3-6METs and Borg scale (score 12–14 or 40–60% of the age-adjusted maximal heart  rate. | | each day for at least 20 min per session (or at least two 10-min  sessions to reach a maximum of 20–30 min)  walking- at least 20–30 min per session (not  including the 5 min of warm-up and 5 min of cool-down) the accumulation of 10-min sessions that will reach a  maximum of 20–30 min. | | 3-5days/week for 12 weeks |
| Chen et al.,2016 | lung cancer/ all stages | >18 years/ 24M + 32 F  111: EX (56) + UC (55) | Surgery (30), Chemotherapy (2), Radiotherapy (2), Target therapy (3), No treatment (19) | EX: home based walking program  UC: The patients were asked to maintain  normal daily activity and not perform additional exercise. | | | moderate intensity | | 40min/session | | 3days/week for 12 weeks (6 months follow up) |
| Roveda et a., 2017 | breast cancer/ no stage specified | 35-70years/ 0M +40F  40: IG (19) + CG (21) | Post-surgical and not on any treatment | IG: Brisk walking  CG: WCRF/AICR recommendation of 30-minute physical activity every day | | | HRmean - 58% to 73% | | 1 hour session | | 2days/week for 3 months |
| Mercier et al., 2018 | all cancer and stages | 18-80years/ 9M+ 32F  41: EX (20) + (21 CBT-I) | Post Cancer treatments:  Surgery  (16(, Radiotherapy (21), Chemotherapy (9), Brachytherapy (1), Current  Hormone Therapy (1) | EX: Home-based Choice of brisk walking, jogging, swimming, or combination.  CBT-I: self-administered treatment package of a 60 minute of video and six booklets. | | | moderate intensity | | 20-30min/session  (150min/week) | | 3-5days/week for 6 weeks (follow up at 6 months) |
| Khoirrunnisa et al., 2019 | blood and solid cancers/ all stages | 8-18 years/ 36M +28 F  64: IG (32) + CG (32) | Chemotherapy | IG: AeRop exercise consists of aerobic exercise and progressive muscle relaxation (PMR).  CG: standard care | | | NR | | 15 min in 5 days | | 4 weeks |
| RESISTANCE TRAINING | | | | | | | | | | | |
| Steinndorf et. al, 2017 | Breast cancer/ stage I–III | >18years  160(F)  160  (80 EG+ 80 RC) | Radiotherapy | EG- Supervised machine based progressive resistance exercise  RC- Jacobson progressive relaxation | | | 60–80% of 1RM  8 exercises x 3sets x 8-12 reps | | 1hour/ session | | 2days/ week for 12 weeks (follow up 12 months) |
| COMBINED TRAINING STUDIES | | | | | | | | | | | |
| Sprod et. al, 2010 | breast and prostate cancer | >18years (11M + 27F)  38: 19 (EG) +19 (CG) | Radiotherapy | 1 instructional session, followed by home based progressive walking + resistance band exercise  CG- instructed to be active as they were prior to the study | | | Walking- moderate  (RPE- 3-5)  Resistance training- low to moderate | | 1^st^ session 45min/ day | | 7days/ week for 4 weeks |
| Chivelle et. al, 2014 | Lung and Colorectal Ca, stage 4 | >18yrs (25M+41F)  66 (33EX + 33) | Chemoradiotherapy | REST (5 resistance exercise routines*2 sets) with instruction + pedometer-based walking. Followed bimonthly by telephone calls for reviewing and advancing the programs  CG- Chemoradiotherapy | | | Moderate | | 90-minute/ session | | REST- At least 2 sessions/ week for 8 weeks  Walking- at least 4 days/ week |
| Kampshoff 2015 | Breast, colon, ovarian, cervix, testis cancer or lymphomas/ stage 1-4 | ≥18 years (77M + 402F)  479 (HI-91, LMI-95, WLC- 91) | Post chemotherapy | Resistance exercises under supervision-  HI and LMI- six resistance exercises with a frequency of 2 sets of 10 repetitions each, targeting large muscle groups  Endurance - static cycling  HI group-  30 seconds at 65 % of MSEC and 60 seconds at 30 %  LMI group-  30 seconds at 45 % of the MSEC and 60 seconds at 30 %.  After 4 weeks the duration of the latter block was reduced from 60 to 30 seconds in both  HI and LMI advised to be physically active at home  WLC- Waitlist control | | | Resistance-  HI- 70-85 % of 1RM  LMI- 40-55% of 1RM  Endurance-  HI- ≥80 % of HRR  LMI- 40–50 % of HRR  Moderate intensity | | NR  30min/ session | | 2days/ week for 12 weeks  3days/ week |
| Rogers et. al, 2015 | breast cancer/ ≤ Stage II | 30 to 70 years/ 42(F)  42: 42 (20-intervention + 22 control) | >4 weeks post primary treatment but on anti-oestrogen agents | Aerobic- unsupervised walking + supervised  Resistance- resistant bands exercises +  6 discussions to improve adherence  Control- asked to avoid changing of exercise behaviour | | | aerobic- moderate intensity  Resistance- 8 exercises x 2 sets x 15 reps | | Aerobic- 40min/ session | | Aerobic- 4days/ week  (2 supervised + 2 home based) for 3 months  Resistance- 2days/ week |
| Coleman 2012 | Multiple myeloma | 35-76 years  95 (EG) + 92 (CG) | Chemotherapy | Aerobic walking + resistance exercises- (a) daily stretching exercises (b) strength and resistance training- biceps curls, triceps extensions (chair push-ups), chair stands and hamstring strengthening) + instructed to remain active and walk  UC- instructed to remain active and walk 20min/day, 3 times/ week | | | Aerobic walking 65%−80% of HRmax (11-13 RPE)  Strength resistance training-60%− 80% of 1RM (15-17 RPE) | | Walking- 20min/day | | 6days/ week with aerobic and resistance on alternate days for 15 weeks |
| Courneya et al.,2014 | breast cancer/ stage I–IIIc | >18years (0M +296 F)  N = 296 (STAN- 95, HIGH- 99, COMB- 102) | Chemotherapy | STAN- vigorous aerobic exercise  HIGH- Vigorous aerobic exercise  COMB- Vigorous aerobic + strength training- 9 exercises x 10-12 reps | | | STAN- Vigorous  HIGH- vigorous  COMB- vigorous aerobic + moderate strength (60–75 % of 1 RM) | | STAN- 25–30 min/ session  HIGH- 50–60 min/ session  COMB- 50–60 min/ session | | STAN- 3 days/week for 3-4 weeks after chemotherapy  HIGH- 3 days/week  COMB- STAN- 3 days/week + strength 3 days/week |
| PHYSICAL ACTIVITY | | | | | | | | | | | |
| Rogers et al., 2009 | breast cancer, stage I, II, or IIIA | 18 and 70 years.  41: 20 (UC) + 21 (EX) | Hormonal therapy | EX: group and individual sessions consisting of 12 individual supervised exercise and 3 individual ‘‘face-to face’’ update counselling sessions with an exercise specialist  that tapered to a home-based program by the end of the intervention.  UC: written materials  related to physical activity obtained from the American  Cancer Society | | | moderate | | 150mins/ week | | For 12 weeks |
| Donnelly et al., 2011 | stage 1-3 of Ovarian,  Endometrial,  Uterine, Cervical | 18years.  33(F).  33: PA (16) + UC (17) | Post-surgical (33), Chemotherapy (13), Radiotherapy (7), Chemotherapy and Radiotherapy (13) | PA: home-based physical activity intervention including walking and strengthening  exercises. Initial consultation with a physiotherapist, followed by weekly telephone calls for 10 weeks and final consultation at 12^th^ week and 2 monthly follow up call.  UC: standard care | | | moderate intensity | | 30 min of physical activity | | at least 5 days of the week |
| Rogers et al., 2013 | Stage I, II, or IIIA Breast Cancer | 18 and 70 years; 28(F).  28: PA (15) + UC (13) | No ongoing Chemotherapy or Radiotherapy; >8 weeks post-surgery | PA: exercise and  resistance training (i.e., up to 20 repetitions of 8 different exercises  using each of the major muscle groups). Participants were tapered from supervised exercise  sessions with an exercise specialist to home-based, unsupervised exercise over the first six  weeks of the intervention and given heart rate monitors to achieve target exercise intensity.  UC: written material from American Cancer Society. | | | moderate intensity | | 150  weekly minutes exercise and two sessions per week of resistance training | | 6 weeks |
| Rogers 2017 | breast cancer/ stage I-IIIA | 18-70 years 222(F)  222: BEAT Cancer (110) + UC (112) | No ongoing Chemotherapy or Radiotherapy; >8 weeks post-surgery | BEAT Cancer: physical activity included 12 supervised exercise sessions with exercise specialists during the first six  weeks that were tapered to entirely unsupervised exercise off-site (e.g., home-based)  supported by update counselling sessions with exercise specialists every two weeks.  UC: written material from American Cancer Society. | | | moderate-to-vigorous | | ≥150 weekly minute | | 3 months |
| Li 2022 | All/stage 1-3 | 15-39yrs  143: 47 (PAG)+ 48 (BAG)+ 48 (CG) | Undergoing/completed surgery, radiotherapy or chemotherapy | PAG- intelligent sports  bracelet and an exercise instruction manual given+ group interaction  BAG-received 120-180min online video seminar once a week in groups of 6-10 + group interaction.  CG- usual treatment & follow up | | | moderate | | 150min/ week | | 8 weeks |
| YOGA | | | | | | | | | | | |
| Cohen et al.,2004 | Lymphoma.  all stages | >18years  38: 19(TY) +19(WL) | Undergoing/completed chemotherapy | TY:  1) controlled breathing & visualization  2) mindfulness 3) postures from the Tsa lung  4) preliminary set of postures (instructed and audiotape given)  WC- no contact with research personnel | | |  | | NR | | 1 session/ week for 7 weeks |
| Raghavendra et al., 2009 | breast/ stage 2 and 3 | 30-70 years.  88 (F).  88: 44(yoga) + 44(supportive counselling) | Radiotherapy | | 1) set of asanas  2) breathing exercises,  3)pranayama  4)meditation  5) yogic relaxation techniques with imagery  CG- received 3-4 counselling session |  | | 60min/ session | | 3 sessions per  Week for 6 weeks | |
| Mustain et al., 2013 | mixed/all | 54 years (0.51).  393F+ 17M.  410: 206(yoga)+ 204 (SC) | Post-surgery, post chemotherapy,  and/or post-radiation therapy. | | YOCAS:  1)standard sitting and standing sequences  2)transitions  3)restorative yoga  4) mudras  5) pranayama  6) mindfulness meditation  7)visualization  CG- received standard follow-up care+ offered YOCAS after the study |  | | 75min/ session | | twice a week for 4 weeks | |
| Chandwani et al.,2014 | breast/ stage 0 to 3 | 26-79years; 178F  178: 53 (Yoga) + 56 (Stretch) + 54 (waitlist) | Radiotherapy | | Yoga-  1) Asana  3) deep relaxation  4) pranayama  5) meditation  ST-  Standing, lying down, sitting positions and approximated the gross movements of the YG exercises. Participants learned all the material over the course of the first four classes taught by physiotherapists.  WLC- Waitlist control |  | | 60min | | 3 sessions/ week for 6 weeks  (Follow up- 6months) | |
| Cramer et al., 2016 | non-metastatic colorectal cancer (stage I–III) | 40-87 years; 21 (F)  33 (M)  54: 27 (Yoga) + 27 (Control) | 2-48 months post-surgery | | Yoga- traditional hatha yoga led by certified hatha yoga instructors  CG- Waitlist control |  | | 90 mins | | weekly for 10 weeks | |
| Taylor et al., 2018 | breast/ no stage mentioned | 18- 65years.  26 (F)  26: 14 (yoga) + 12 (control) | Post treatment | | Restorative yoga:  1) 10 min of Pranayama  2) 5 min of meditation in cross-legged sitting  3) 50 min of 13 restorative yoga postures of active relaxation  4) 10 min of sav asana  CG- maintained daily activities and didn’t participate in yoga for 8 weeks |  | | 75 min | | 1 session/ week for 8 weeks | |
| Chaoul et al.,2018 | breast/ 1 to 3 stages | >18years.  227 (F)  227: 74 (TYP) +68 (STP) + 85(UC) | Chemotherapy | | TY:  1) mindfulness and focused attention through guided meditation with breathing & visualization  2) Alternate nostril breathing practice & breath retention exercise  3) Tsa Lung movements  4) compassion-based  meditation.  STP:  Included stretches in standing, lying down, and sitting positions and approximated the gross movements of the TYP  CG- received no exercise/yoga |  | | 75-90 min | | 4 supervised sessions during chemotherapy, and 3 booster sessions over the subsequent 6 months + encouraged to practice at  home 7days/week | |
| Huberty et. al, 2019 | Myelo-proliferative neoplasm  (Thrombo-  cythemia, polycythemia vera, or myelofibrosis) | >18years.  3(M) + 45 (F)  48: 27 (yoga) + 21 (control) | Completed treatment | | Yoga- home based, online streamed videos include warm-up and cool down, reminders for breathing with the movements/ poses, and a closing mindfulness activity  with a message from yoga instructor, brief meditation, and final relaxation. |  | | Week 1–2  brief introductory videos (5–20 min)  Week 3–12  (20–30 min) with slightly increased intensity | | 5-30min/ session  60min/week for 12 weeks (follow up till 16 weeks) | |
| TAI-CHI | | | | | | | | | | | |
| Larkey et. al, 2015 | breast cancer survivors/ Stage 0-III | 40–75 years.  87 (F).  87: 87 (SQG- 45 +  QG/TCE- 42) | >6 months post treatment | | QG/TCE-quigong/tai chi for 12 weeks by nurse. Followed up at 3 months  SQG- quigong taught by exercise physiologist | Low | | 30min/session  supervised 60min/day | | 5days/week  supervised 2days/week | |
| Irwin et. al, 2017 | breast cancer survivors/ Stage 0-III | 42 to 83 years 90(F)  90 :(45 TCC + 45 CBT-I) | >6 months after completion of treatment | | TCC- group of 7-10 over  2 months +1 month of skill consolidation + adherence for 3 months  CBT-I- group of 7-10 for 120min/ week | NR | | 120min/ week | | NR | |
| McQuade 2017 | rectal, anal, prostate cancer/ 1-3 stages | >18years; 76(M)  90: QGTC (26) + LE (26) + WLC (24) | Radiotherapy | | QGTC: by trained master, in groups of 1- 2 patients. Included- preparation exercises + main exercises + ending exercises.  Also, DVD and instruction material was given to practice daily.  LE- given by exercise physiologist. effects. Had combination of 3 levels of resistance tubes and the focus was on lighter tension and more repetition (8–12 per set) + stretching  WLC-Waitlist control | NR | | 40min/session | | 3days/week supervised + 4days/ week unsupervised | |
| Lu et. al., 2019 | Colorectal cancer/ stages 1-3 | >18yrs  56 (M)  87: 43 (BEG) + 44 (CG) | Undergoing chemotherapy, post surgery | | BEG- given course videos and educated regarding the benefits. During hospitalisation, sessions led by nurse. During home stay, participants asked to perform and send picture of logs on Wechat app.  CG- usual care |  | | 20-40min/ session, 5days/week | | 24 weeks | |

*AET- Aerobic Exercise Training; BEAT Cancer- Better exercise adherence after treatment for cancer*;  *BEG-Baduanjin exercise GROUP ; BAG- Behavioral activity group, CBT-I- cognitive-behavioural therapy for insomnia intervention group; CC- received usual care throughout the treatment; CG- Control group; CE- received exercise prescription and regular follow-up after completing the cancer treatment- Control Group; COMB-combination exercise; HI- high intensity, HIGH- high intensity aerobic exercise; EE-received exercise prescription and follow-up throughout the study protocol; EG- Exercise Group;* *EX- Exercise Intervention;* *IG - Intervention Group; LE- light exercise; LMI- low moderate intensity; NR-Not Reported; PA: Physical Activity; PAG- Physical activity group; QGTC or TCC or* *QG/TCE- Quigong/ tai chi exercise; RC- Relaxation control group; SQG- sham quigong; STAN- Standard aerobic exercise; ST-Stretching; STP-stretching program; TY-Tibetian Yoga; TYP- tibetian yoga program; UC-Usual care; UG- Usual care Group; WLC- Waitlist control;* YOCAS-*yoga for cancer survivors; MFI-Movement & Fragmentation Index; PSQI- Pittsberg Sleep Quality Index; r24- 24 hours autocorrelation coefficient; SDS-Sleep Disturbance Scale; SE-Sleep Efficiency; SOL-Sleep Onset Latency; TST- Total Sleep time; WASO- Wake after sleep onset*
