# Supplementary material for: Effect of exercise based interventions on sleep and circadian rhythm in cancer survivors—a systematic review and meta-analysis
Source: PeerJ. 2024 Mar 8;12:e17053. doi: 10.7717/peerj.17053 (PMC10926908; doi:10.7717/peerj.17053)
Supplement: Supplemental Information 11 [file peerj-12-17053-s011.docx]

**S-4-** Summary of results of the exercise studies.

| Author, Year | Results | | |
| --- | --- | --- | --- |
| **AEROBIC TRAINING** | | | |
| Payne et. al, 2008 |  | Pre- mean (SD) | Post- mean (SD) |
|  | **PSQI** | | |
|  | EX | 13 | 11* |
|  | UC | 8 | 9 |
|  | **Accelerometer** | | |
|  | Actual wake time (minutes) | | |
|  | EX | 0.4 | 0.5* |
|  | UC | 0.9 | 1.3 |
|  | Actual sleep time (minutes) | | |
|  | EX | 5.6 | 5.6 |
|  | UC | 5.4 | 6.4 |
|  | Movement in sleep | | |
|  | EX | 58 | 58* |
|  | UC | 90 | 120 |
|  | Sleep efficiency (%) | | |
|  | EX | 82 | 82 |
|  | UC | 78 | 80 |
|  | **Biomarkers (for Circadian rhythm)** | | |
|  | Serum cortisol (mcg/dl) | | |
|  | EX | 9 | 8 |
|  | UC | 9.5 | 8 |
|  | Serum serotonin (pg/dl) | | |
|  | EX | 140 | 80* |
|  | UC | 90 | 130 |
| Tang et. al, 2009 | **PSQI** |  |  |
|  | EX | 13.42 (2.27) | 9.78 (3.06)* |
|  | UC | 13.17 (3.11) | 13.11 (2.89) |
| Dodd et. al, 2010 | **PSQI** |  |  |
|  | EX | 45 | 40 |
|  | UC | 43 | 43 |
| Wang et al., 2011 | **PSQI** | | |
|  | EG | 9.14 (4.70) | 7.50 (3.20) * |
|  | UG | 9.32 (4.30) | 9.32 (4.30) |
| Courneya et al., 2012 | **PSQI** | | |
|  | AET | 5.84 (3.58) | 4.47 (3.13) |
|  | UC | 4.15 (3.04) | 4.15 (3.19) |
| Cho et al., 2012 | **GSDS** | | |
|  | Exerciser | 44.95 (17.93) | 44.84 (20.07) |
|  | UC | 47.49 (20.53) | 43.42 (19.42) |
| Wenzel et al., 2013 | **PSQI** | | |
|  | EX | 6.7 (3.9) | 7.7 (4.2) * |
|  | UC | 5.8 (3.7) | 6 (3.5) |
| Naraphong et al., 2015 | **GSDS** | | |
|  | EG | 3.45 (1.14) | 3.87 (1.61) |
|  | CG | 3.75 (1.39) | 3.78 (1.27) |
| Chen et al., 2016 | **PSQI** | | |
|  | EX | 9.25  (4.55) | 6.49*  (3.71) |
|  | UC | 8.82  (4.26) | 8.33  (4.67) |
|  | **Accelerometer** | | |
|  | Total sleep time (minutes) | | |
|  | EX | 380.32 (96.39) | 401.76 (72.84) * |
|  | UC | 395.06 (88.21) | 395.06 (88.21) |
|  | Wake after sleep onset (minutes) | | |
|  | EX | 45.86 (33.17) | 52.61 (43.96) |
|  | UC | 50.56 (45.30) | 63.92 (66.42) |
|  | Sleep onset latency (minutes) | | |
|  | EX | 27.14 (40.48) | 22.15 (23.00) |
|  | UC | 27.14 (40.48) | 37.88 (38.05) |
|  | Sleep efficiency (%) | | |
|  | EX | 88.94 (9.67) | 88.18 (10.78) |
|  | UC | 88.36 (10.73) | 85.07 (15.38) |
|  | r24 | | |
|  | EX | 0.42 (0.14) | 0.47 (0.15) |
|  | UC | 0.36 (0.12) | 0.41(0.18) |
|  | I < O | | |
|  | EX | 94.68 (7.31) | 96.63 (2.98) |
|  | UC | 92.65 (7.24) | 93.45 (8.30) |
| Roveda et al., 2017 | **Accelerometer** | | |
|  | Actual wake time (minutes) | | |
|  | IG | 55 (22.5) | 52.6 (20.5) * |
|  | CG | 49.9 (20.4) | 60.3 (20.4) |
|  | Actual sleep time (minutes) | | |
|  | IG | 392.6 (42.4) | 386.1 (42.2) * |
|  | CG | 402.1 (37.5) | 374.1 (40.3) |
|  | Immobility time (minutes) | | |
|  | IG | 88.4 (3.5) | 87.1 (4.4) * |
|  | CG | 87.8 (3.6) | 83.7 (5.3) |
|  | Sleep efficiency (%) | | |
|  | IG | 84.4 (4.3) | 84.6 (4.9) * |
|  | CG | 85.2 (5.2) | 80.7 (6.0) |
|  | Mean activity score | | |
|  | IG | 17.7 ± 7.6 | 15.9 ± 6.8* |
|  | CG | 13.7 ± 5.2 | 19.2 ± 7.1 |
|  | Movement & fragmentation Index | | |
|  | IG | 25.0 ± 7.1 | 27.4 ± 8.0* |
|  | CG | 27.2 ± 9.4 | 31.7 ± 10.6 |
|  | Sleep latency (minutes) | | |
|  | IG | 11.7 (11.1) | 15.4 (9.5) * |
|  | CG | 15.7 (10.0) | 25.1 (18.5) |
|  | MESOR |  |  |
|  | IG | 232.9 (SE 18.1) | 221.9 (SE 10.8) |
|  | CG | 223.7 (SE 14.5) | 215.8 (SE 9.5) |
| Mercier et al., 2018 | **PSQI** | | |
|  | EX | 11.7 (0.9) | 7.6 (1.0) |
|  | CBT-I | 12.1 (0.8) | 9.3 (0.8) |
|  | **Insomnia Severity Index** | | |
|  | EX | 16.0 (1.3) | 11.8 (1.2) |
|  | CBT-I | 14.8 (1.1) | 9.4 (1.3) |
|  | **Accelerometer** | | |
|  | Sleep onset latency (minutes) | | |
|  | EX | 39(7) | 20.6 (5.2) |
|  | CBT-I | 34.7 (4.5) | 20.4(3.1) |
|  | Wake after sleep onset (minutes) | | |
|  | EX | 63.2 (11.7) | 18.3 (5.8) |
|  | CBT-I | 56.9 (9.4) | 30.6 (4.3) |
|  | Sleep efficiency (minutes) | | |
|  | EX | 71.3 (3.4) | 85.4 (1.9) |
|  | CBT-I | 73.8 (2.6) | 84.1 (1.7) |
|  | Total sleep time (minutes) | | |
|  | EX | 384.1 (17.9) | 448.7 (8.8) |
|  | CBT-I | 380.4 (14.7) | 428.3 (14.3) |
| Khoirrunnisa et al., 2019 | **Sleep Disturbance Scale for Children** | | |
|  | IG | NR | 33.69(3.03) * |
|  | CG | NR | 32.09  (3.14) |
| **RESISTANCE TRAINING** | | | |
| Steinndorf et. al, 2017 | **EORTCQLQ-C30 insomnia subscale (sleep)** | | |
|  | Hours slept during night (hours) | | |
|  | EG | 6.6 (1.2) | 6.6 (1.3) |
|  | RC | 6.5 (1.6) | 6.7 (1.4) |
|  | Number of awakenings at night | | |
|  | EG | 2.0 (1.3) | 2.1 (1.3) * |
|  | RC | 2.4 (1.5) | 2.4 (1.4) |
|  | Napping during the day (minutes) | | |
|  | EG | 30 (0, 45) | 15 (0, 30) * |
|  | RC | 15 (0, 40) | 0 (0, 33) |
| **COMBINED TRAINING** | | | |
| Sprod et. al, 2010 | **PSQI** | | |
|  | EG | 7.06 (4.26) | 6.00 (3.87) |
|  | CG | 7.79 (4.00) | 7.44 (4.72) |
| Chivelle et. al, 2014 | **11-point NRS** | **Mean difference (SD)** | |
|  | REST | 1.46 (1.88) * | |
|  | CG | −0.10 (1.71) | |
| Kampshoff 2015 | **PSQI** | | |
|  | HI | 10.3 (3.3) | 9.9 (3.3) |
|  | LMI | 10.9 (3.1) | 10.7 (3.7) |
|  | WLC | 10.1 (3.2) | 9.9 (3.6) |
| Rogers et. al, 2015 | **PSQI** | | |
|  | EG | 8.2 (3.4) | 6.7 (3.7) |
|  | CG | 9.2 (4.8) | 7.1(3.2) |
|  | **Accelerometer** | | |
|  | Sleep Efficiency (%) | | |
|  | EG | 82.3 (6.2) | 82.9 (5.7) |
|  | CG | 82.4 (8.6) | 84.9 (6.0) |
|  | Sleep Onset Latency (minutes) | | |
|  | EG | 10.3 (10.5) | 7.4 (5.6) |
|  | CG | 7.1 (6.8) | 8.9 (6.6) |
| Coleman et al., 2012 | **Accelerometer** | | |
|  | Sleep Efficiency (%) | | |
|  | EG | 79.7 (15) | 81.39 (14.01) |
|  | CG | 77.79 (18.96) | 76.57 (20.51) |
| Courneya et al., 2014 | **PSQI** | | |
|  | STAN | 6.14 (0.31) | 7.61 (3.91) |
|  | HIGH | 6.23 (4.35) | 6.71 (0.31) * |
|  | COMB | 6.22 (4.07) | 6.86 (0.30) * |
| **PHYSICAL ACTIVITY** | | | |
| Rogers et al., 2009 | **PSQI** | | |
|  | EX | 6.20 (3.94) | 6.65 (4.17) |
|  | UC | 5.22 (3.57) | 5.50 (4.00) |
| Donnelly et al., 2011 | **PSQI** | | |
|  | PA | 8.94 (3.92) | 7.19 (4.20) |
|  | UC | 9.76 (4.13) | 10.18 (4.98) |
| Rogers et al., 2013 | **PSQI** | | |
|  | PA | 6.5 (3.1) | 6.3 (2.7) |
|  | UC | 5.4 (3.4) | 6.2 (3.2) |
|  | **Accelerometer** | | |
|  | SE (%) | | |
|  | PA | 81.4 (10.3) | 79.5 (8.3) |
|  | UC | 80.4 (9.9) | 83.2 (10.5) |
|  | SOL (minutes) | | |
|  | PA | 8.7 (6.9) | 7.1 (3.8) |
|  | UC | 9.3 (5.0) | 9.8 (5.2) |
| Rogers et al., 2017 | **PSQI** | | |
|  | BEAT Cancer | 8.5 (3.7) | 7.3 (3.8) * |
|  | UC | 7.3 (4.1) | 7.6 (4.2) |
|  | **Accelerometer** | | |
|  | \| SE (%) \| \| --- \| | | |
|  | BEAT Cancer | 83.2 (5.8) | 82.6 (7.2) |
|  | UC | 81.6 (7.0) | 81.9 (6.9) |
|  | \| SOL (minutes) \| \| --- \| | | |
|  | BEAT Cancer | 8.9 (7.5) | 8.6 (7.3) |
|  | UC | 9.9 (8.2) | 10.2 (10.3) |
| Li et. al., 2022 | **PSQI** | | |
|  | PAG | 6.32 (3.108 ) | 4.86 (2.339)* |
|  | BAG | 6.46 (2.858) | 5.05 (1.591) |
|  | CG | 6.42 (2.850) | 5.88 (2.303) |
| **YOGA** | | | |
| Cohen et al., 2004 | **PSQI** | | |
|  | TY | 6.5 (5.0) | 5.8 (2.3) * |
|  | WC | 7.2 (4.7) | 8.1 (2.4) |
| Raghavendra et al., 2009 | **Salivary cortisol (Circadian rhythm)** | | |
|  | YOGA | 0.25 (0.13) | 0.19 (0.13) * |
|  | CG | 0.25 (0.21) | 0.25 (0.18) |
| Mustain et al., 2013 | **PSQI** | | |
|  | YOCAS | 9.20 (0.25) | 7.23 (0.26) * |
|  | CG | 8.96 (0.28) | 7.89 (0.26) |
| Chandwani et. al, 2014 | **PSQI** | | |
|  | YOGA | 8.3 (0.6) | 6.7 (0.5) |
|  | UC | 8.2 (0.5) | 7.3 (0.5) |
|  | **Salivary cortisol (Circadian rhythm)** | | |
|  |  | Mean difference -0.104 | SE 0.011* |
| Cramer et. al, 2016 | **PSQI** | | |
|  | YOGA | 9.11 ± 3.27 | 9.21 ± 2.14* |
|  | UC | 9.88 ± 2.58 | 10.34 ± 2.87 |
| Taylor et al., 2018 | **ISI** | | |
|  | YOGA | 10.18 (8.74) | 7.89 (7.17) |
|  | CG | 7.56 (6.82) | 6.20 (7.11) |
| Chaoul et al., 2018 | **PSQI** | | |
|  | TY | 7.8 (3.7) | 6.3 (4.1) |
|  | STP | 8.5 (3.9) | 6.5 (3.4) |
|  | CG | 8.1 (4.2) | 6.1 (3.9) |
|  | **Accelerometer** | | |
|  | SE (%) | | |
|  | TY | 81.2 (6.7) | 81.6 (5.5) * |
|  | STP | 80.7 (6.6) | 82.0 (4.9) |
|  | CG | 81.4 (6.1) | 83.3 (3.8) |
|  | SOL (minutes) | | |
|  | TY | 34.4 (22.2) | 40.0 (26.4) |
|  | STP | 29.5 (17.9) | 31.8 (20.2) |
|  | CG | 32.9 (19.2) | 34.1 (18.0) |
|  | TST (hours) | | |
|  | TY | 7.2 (0.8) | 7.3 (0.8) |
|  | STP | 7.1 (1.1) | 7.3 (1.1) |
|  | CG | 7.1 (1.1) | 7.2 (0.9) |
|  | WASO (minutes) | | |
|  | TY | 44.0 (25.4) | 39.9 (8.9) * |
|  | STP | 48.4 (17.1) | 45.5 (15.7) * |
|  | CG | 48.8 (21.2) | 44.6 (16.3) |
| Huberty et. al, 2019 | **Sleep Disturbance Short Form 8a (sleep)** | | |
|  | YOGA | 50.1 (6.2) | -2.3 (5.8) |
|  | CG | 48.8 (6.7) | 0.2 (5.2) |
| **TAI-CHI** | | | |
| Larkey et. al, 2015 | **PSQI** | | |
|  | QG/TCE | 10.0 (3.85) | 6.3 (3.00) |
|  | SQG | 9.9 (4.77) | 7.7 (4.94) |
| Irwin et. al, 2017 | **PSQI** | | |
|  | TCC | 11.5 (0.5) | 7.4 (0.5) |
|  | CBT-1 | 11.2 (0.4) | 6.8 (0.4) |
|  | **Diary-SOL** | | |
|  | TCC | 37.1 (2.7) | 25.7 (3.3)* |
|  | CBT-1 | 41.1 (2.7) | 14.4 (2.9)* |
|  | **Diary -SE** | | |
|  | TCC | 71.4 (1.4) | 80.6 (1.7) * |
|  | CBT-1 | 70.8 (1.4) | 86.0 (1.5) * |
|  | **Diary- WASO** | | |
|  | TCC | 56.0 (3.4) | 39.9 (4.1) * |
|  | CBT-1 | 52.2 (3.5) | 28.4 (3.8) * |
| McQuade et al., 2017 | **PSQI** | | |
|  | QGTC | 6.85 (0.76) | 5.16 (0.52) |
|  | LE | 5.58 (0.78) | 5.33 (0.63) |
|  | WLC | 6.58 (0.69) | 5.77 (0.50) |
| Lu et. al., 2019 | **PSQI** | | |
|  | BEG | 10.5 (1.5) | 4.1 (1.1) * |
|  | CG | 10.5 (2.1) | 6.9 (2.0) |

*AET- Aerobic Exercise Training; AST- Actual Sleep Time; AWT-Actual Wake time; BEAT Cancer- Better exercise adherence after treatment for cancer*; *CBT-I- cognitive-behavioural therapy for insomnia intervention group; CC- received usual care throughout the treatment; CE- received exercise prescription and regular follow-up after completing the cancer treatment- Control Group; COMB-combination exercise; EE-received exercise prescription and follow-up throughout the study protocol; EG- Exercise Group;* *EX- Exercise Intervention; GSDS-General Sleep Disturbance Scale;* *HI- high intensity, HIGH- high intensity aerobic exercise; I < O- in-bed less than out-of-bed dichotomy index; IG - Intervention Group; ISI-Insomnia Severity Index; LE- light exercise; LMI- low moderate intensity; NR-Not Reported; PA: Physical Activity; PSQI- Pittsberg Sleep Quality Index; QGTC or TCC or* *QG/TCE- Quigong/ tai chi exercise; r24- 24 hours autocorrelation coefficient; RC- Relaxation control group; SDS-Sleep Disturbance Scale; SE-Sleep Efficiency; SOL-Sleep Onset Latency; SQG- sham quigong; STAN- Standard aerobic exercise; ST-Stretching; STP- stretching program; TY-Tibetian Yoga; TYP-tibetian yoga program; TST- Total Sleep time; UC-Usual care; UG- Usual care Group; WLC- Waitlist control; WASO- Wake after sleep onset* YOCAS*- Yoga for Cancer Survivors*
