# Supplementary material for: Effect of exercise based interventions on sleep and circadian rhythm in cancer survivors—a systematic review and meta-analysis
Source: PeerJ. 2024 Mar 8;12:e17053. doi: 10.7717/peerj.17053 (PMC10926908; doi:10.7717/peerj.17053)
Supplement: Supplemental Information 13 [file peerj-12-17053-s013.docx]

For systematic reviews / meta-analyses, authors need to provide the following information:

1. The rationale for conducting the systematic review / meta-analysis;
2. The contribution that it makes to knowledge in light of previously published related reports, including other meta-analyses and systematic reviews.
3. Cancer survivors are more likely to present with disrupted circadian rhythm not only during- or post-treatment but even prior to the initiation of the primary cancer treatment. This de-regulated circadian rhythm in cancer survivors is associated with a poor quality of life and prognosis, high mortality and sleep disturbances. Sleep disturbance in cancer survivors is a chronic disabling problem with a high prevalence rate ranging from 30% to 50%. The underlying mechanisms contributing to these disturbances are believed to multifactorial, including a host of physical and psychological components. The multifactorial nature of these disturbances in cancer survivors suggests that a single therapeutic approach (with pharmacological approaches typically used initially), may not adequately address all the mechanisms contributing to these issues. In addition, while common pharmacological treatments prescribed for sleep disorders can significantly improve sleep outcomes in cancer survivors, they also have numerous side-effects. Hence, our systematic review and meta-analysis was set to summarise the effect of all forms of exercise interventions of sleep and circadian rhythm in cancer survivors.
4. Modes of exercise that have been examined in cancer survivors include general physical activities such as walking, as well as more structured exercise modes including aerobic exercise, resistance exercise, yoga and tai chi. Although there is some evidence suggesting that exercise may potentially improve certain aspects of sleep among cancer survivors, these reviews still have their limitations. There is lacuna of evidence that encapsulates all forms of exercise, with most studies involving resistance or aerobic exercise common in the Western World compared to other forms of exercise such as yoga and tai chi that originated in Asian countries.

There is also lack of evidence summarizing the effect of exercise on circadian rhythm in cancer survivors.

Further, previous reviews either had no restriction on the study design and included studies other than RCTs or excluded RCTs which had no individual patient data. Hence, this systematic review was developed to summarize RCT level evidence of all forms of exercise on sleep and circadian rhythm outcomes across all groups of cancer survivors.
